# Secondary care provider attitudes towards patient generated health data from smartwatches

**DOI:** 10.1038/s41746-020-0236-4

**Published:** 2020-03-03

**Authors:** Jordan M. Alpert, Todd Manini, Megan Roberts, Naga S. Prabhakar Kota, Tonatiuh V. Mendoza, Laurence M. Solberg, Parisa Rashidi

**Affiliations:** 1 Department of Advertising, College of Journalism and Communications, University of Florida, Gainesville, FL USA; 2 Department of Aging and Geriatric Research, University of Florida, Gainesville, FL USA; 3 Computer & Information Science & Engineering, University of Florida, Gainesville, FL USA; 4 Department of Health Outcomes and Biomedical Informatics, University of Florida, Gainesville, FL USA; 5 Veterans Health Administration, NF/SG VHS, Geriatrics Research, Education and Clinical Center (GRECC), Gainesville, FL USA; 6 College of Nursing, University of Florida, Gainesville, FL USA; 7 J. Crayton Pruitt Family Department of Biomedical Engineering (BME), University of Florida, Gainesville, FL USA

**Keywords:** Health policy, Health policy, Patient education, Patient education

## Abstract

Wearable devices, like smartwatches, are increasingly used for tracking physical activity, community mobility, and monitoring symptoms. Data generated from smartwatches (PGHD_SW) is a form of patient-generated health data, which can benefit providers by supplying frequent temporal information about patients. The goal of this study was to understand providers’ perceptions towards PGHD_SW adoption and its integration with electronic medical records. In-depth, semi-structured qualitative interviews were conducted with 12 providers from internal medicine, family medicine, geriatric medicine, nursing, surgery, rehabilitation, and anesthesiology. Diffusion of Innovations was used as a framework to develop questions and guide data analysis. The constant comparative method was utilized to formulate salient themes from the interviews. Four main themes emerged: (1) PGHD_SW is perceived as a relative advantage; (2) data are viewed as compatible with current practices; (3) barriers to overcome to effectively use PGHD_SW; (4) assessments from viewing sample data. Overall, PGHD_SW was valued because it enabled access to information about patients that were traditionally unattainable. It also can initiate discussions between patients and providers. Providers consider PGHD_SW important, but data preferences varied by specialty. The successful adoption of PGHD_SW will depend on tailoring data, frequencies of reports, and visualization preferences to correspond with the demands of providers.

## Introduction

Consultations in the clinic between patients and providers are the main opportunity to exchange information, discuss treatment options, and develop a trusting relationship^1^. However, patients with chronic issues feel pressure from the time limitations, which prevent them from sharing important details related to their care^2^. As a consequence, clinicians’ understanding of the effects of disease and treatment on patients during their day-to-day lives is poor^3^. Furthermore, optimal evidence-based medicine is challenging because providers’ are usually unaware whether patients are implementing management plans into their daily lives^4^.

Technological advances have the ability to improve healthcare delivery^5^. mHealth technology, like wearable devices, are increasingly used for community mobility, and tracking physical activity and symptoms (e.g., pain)^6,7^. Data generated, otherwise known as patient-generated health data, has received significant attention because of its potential to foster better communication, improve care coordination, and strengthen patient engagement^8–11^. Patient-generated health data can be produced from wearable devices, such as smartwatches (PGHD_SW). Sixteen percent of U.S. adults own a smartwatch^12^, and over 56 million U.S. adults will use a wearable device at least once per month in 2019^13^.

PGHD_SW differs from other types of data, like patient-reported outcomes (PRO) because PRO data are typically standardized surveys informed by healthcare professionals to understand patients’ experiences of health. Patient Reported Outcomes Measurement Information System (PROMIS) are obtained by surveying patients about topics such as fatigue, pain, anger, and satisfaction that follow historical questionnaire formats. PGHD_SW often includes PROs and is patient-directed, collected through commercial tools, such as mobile phone applications and activity trackers^14^. Patient-generated health data, including data from smartwatches, contributes to patient empowerment^15^, helps patients make sense of their disease, enhances trust with providers^16^, and enables autonomy^10^.

PGHD_SW supplies frequent temporal information about patients. Providers typically rely on patients’ recall to track and assess management routines for patients with chronic disease^17^. PGHD_SW contributes to providers’ decision-making^18^ and fosters a deeper and more accurate understanding of a patient’s illness between clinic visits^19^. However, adoption of PGHD_SW has been hindered by provider perceptions that it contributes to information overload, concerns about liability from lack of timely review^20,21^, and fears around patient privacy^10^. It also remains unclear which digital biomarkers are valuable for clinicians, and how they should be visualized^22^.

Providers have expressed favorable attitudes towards behavioral tracking technologies^23^, but preliminary studies have been limited to general sentiment towards PGHD_SW. As smartwatch technology continues to evolve, with the addition of new data points (e.g., heart rate monitoring, electrocardiogram tracking, and blood pressure) and more precise measures of existing health indicators, it is necessary to understand providers’ perceptions and preferences towards the technology. Therefore, the goal of this study was to perform qualitative interviews with various providers to identify the value of specific data points (e.g., physical activity, fatigue), discover effective techniques to present data (e.g., graphical charts or tables), and learn how to practically incorporate PGHD_SW into practice (e.g., frequency of reports).

Diffusion of Innovations (DoI)^24^ was used as a framework, since the theory helps to understand factors that facilitate the adoption of new technology in health care^25^, including wearable devices^26,27^. Perceptions of the attributes of an innovation are a main indicator whether the innovation will be accepted. The five main attributes are: (1) relative advantage; (2) compatibility; (3) complexity; (4) trialability; (5) observability^24^. Using four of these attributes (observability was not yet pertinent since we were only inquiring about perceptions), we conducted in-depth qualitative interviews to answer the following research questions:

1. What are the benefits and barriers to incorporating PGHD_SW into practice?
2. How should PGHD_SW be visually presented and what are the most clinically valuable data points?
3. What are PGHD_SW’s implications on patient-provider communication?

## Results

### Demographic characteristics

Twelve out of 20 providers (60% enrollment rate) gave written consent to participate from October 2018 to August 2019. Interviews averaged 27 min in length. Most participants were male (*n* = 7, 58%) and the average age was 45 years (SD = 9.8). One provider was in his last year of residency, but all other providers averaged 12 years (SD = 9.4) of practice experience after residency. Most providers specialized in gerontology (*n* = 4) and worked exclusively in out-patient settings (*n* = 5; 42%). A full demographic summary is in Table 1.

**Table 1:** Sample characteristics.

Variable	n (%)
Sex	
Female	5 (42%)
Male	7 (58%)
Average Age	45.1
Average Years in Practice	12.4
Race	
White	8 (67%)
Indian	2 (17%)
Latino	1 (8%)
Asian	1 (8%)
Specialty	
Geriatric	4 (33%)
Orthopedic Surgery	4 (33%)
Anesthesiology	2 (17%)
Nursing	1 (8%)
Physical medicine and rehabilitation	1 (8%)
Patient setting	
Out-patient	5 (42%)
In-patient	3 (25%)
Both	4 (33%)
Current smartwatch ownership	
Yes	5 (42%)
No	7 (58%)

### Themes

Four main themes and associated sub-themes emerged: (1) PGHD_SW is perceived as a relative advantage; (2) data are viewed as compatible with current practices; (3) barriers to overcome to effectively use PGHD_SW; (4) assessments from viewing sample data.

#### Theme 1: PGHD_SW is perceived as a relative advantage

Overall, providers were optimistic about PGHD_SW and viewed it as equivalent to other types of medical data. Five (42%) providers were familiar with similar data, such as PROMIS measures, and three (25%) had experience using such data. Providers acknowledged the potential advantages of PGHD_SW, such as revealing insights about patients that they would typically not be able to access. For instance, referring to measures like pain and physical activity, a surgeon said, “We don’t have a way of getting this stuff when patients are at home…It’s the biggest void in healthcare right now”. Another surgeon who had experience with PROMIS viewed PGHD_SW as extremely valuable and stated:

“We have historically put more stock into the stuff we generate, like blood tests, physical exam findings. But there is a definite value in paying attention to what the patient is generating…I pay a lot more attention to that stuff than I do to their vital signs, honestly.”

All but one provider echoed this sentiment and considered PGHD_SW as commensurate with traditional types of medical data. The lone dissenter, an anesthesiologist, did not see as much value in PGHD_SW due to typically caring for patients in intensive care or emergency situations.

##### Theme 1a: Stimulates in-person communication

Aside from emergency situations, the same anesthesiologist acknowledged that PGHD_SW would help to keep him abreast of a patient’s status. He provided a hypothetical scenario: “I would start the conversation with ‘I see very high pain scores. I see you’re not moving a lot. I see we got an alert here. Did you fall?’ I think that’s a good starting point rather than ‘how are you?’”. Other providers appreciated the ability to use the data to enhance the consultation and provoke more productive discussions. A geriatrician preferred to review the data before the consultation, and then implement a new part of their care plan based on the data. Surgeons viewed PGHD_SW as a means to re-connect with patients after discharge. Referring to a recently discharged patient who might be struggling, an orthopedic surgeon envisioned:

“We [would] say, ‘please come in we need to talk. What’s going on? Why are you struggling?’ We show them that data. ‘You’re only walking 20 feet. The average for all our patients has been this much.’”

##### Theme 1b: Activation

In addition to using PGHD_SW as a platform to communicate with patients, some providers appreciated the ability to show patients data points. A nurse said, “[The data] can be powerful if patients can own it and take control”. Similarly, a surgeon suggested that the data can be used to form partnerships, enabling honest dialogue. The surgeon imagined a situation in the in-patient setting where data could be used to corroborate feedback from both nurses and the patient:

[I could] ask the nurse, ‘how much did the patient get up today? The patient got up twice today.’ Then, you look at [the data] and they actually moved for three minutes. I’d be like, ‘hey look, you haven’t been moving today. Look at the graph.’

#### Theme 2: Data are viewed as compatible with current practices

Providers from various specialties had different viewpoints about which data to use and how to use it. Few were concerned about possible ramifications of increased workload, and instead, believed that PGHD_SW may create efficiencies. Two-thirds (67%; *n* = 8) of providers did not express fears of data overload. This lack of concern was prefaced with the assumption that providers would have control over the frequency of reports. A geriatrician clarified that a monthly report may be too frequent, but a quarterly report would be helpful. An orthopedic surgeon was excited by the data and considered additional data, “Not a nuisance, that’s actually more convenience”.

All 12 (100%) participants believed that data reports should be integrated with the electronic medical record (EMR). Not only is the EMR a secure environment, but it is the most convenient method of viewing information since everything about the patient is consolidated in one place. A rehabilitation physician suggested that reports come as a secure message, which would initially be reviewed by a nurse. Since providers regularly interact with the EMR, they can instantly be made aware of incidents that need their attention, like a fall. Similarly, a surgeon stated, “I would most want push notification if you could set parameters on worrisome trends”.

##### Theme 2a: Data preferences differ by specialty

Each medical specialty required different types of data and had different uses for the data. For example, PGHD_SW reports and their frequency would differ greatly among geriatricians caring for older individuals in the out-patient setting than surgeons or anesthesiologists taking care of in-patients after undergoing an operation. A surgeon conjectured about a scenario that might occur after conducting knee surgery. He said, “We typically bring in all our knees at two weeks, but if we can get daily reports for the first ten days, we can say ‘you don’t need to come in, you’re fine’”. An anesthesiologist preferred PGHD_SW delivered six times a day, but a nurse overseeing chronic disease patients said that monthly reports are beneficial to provide a “good picture” when patients come back for an appointment in 6 months. Specific data points also varied by specialty, which is discussed in theme four.

##### Theme 2b: Trust

Nine (75%) providers would trust PGHD_SW just as much as information directly from questioning patients. A surgeon summarized this sentiment by saying, “If the patient is reporting a high amount of pain, I have to trust what the patient says”. However, three providers (25%) were skeptical about the validity of PGHD_SW. The rehabilitation physician and nurse both thought the data, such as physical activity, can be easily manipulated and patients may skew the data by entering information that makes them “look good”. Likewise, a geriatrician was doubtful about patients entering information about medication adherence. She said, “I think people are not often truthful about it, so I don’t know if a daily report would be helpful”.

#### Theme 3: Barriers to overcome to effectively use PGHD_SW

Less than half (*n* = 5; 42%) of providers owned a smartwatch, but all were generally familiar with their functionality. Providers without a smartwatch, or those with no experience ever using one, expressed some trepidation about incorporating them into their practice. This reaction was encapsulated by an orthopedic surgeon who said, “Would I be comfortable [educating patients]? Yes. Is it a good use of my time? Probably not”. Only one provider, an anesthesiologist, was opposed to devoting any amount of time to discuss smartwatch functionality with patients, saying, “You run the risk of physicians becoming IT support”.

#### Theme 4: Assessments from viewing sample data

Providers reviewed a list of data points (e.g., falls, mood, cognition tests, and physical activity) and selected the three attributes that they considered most and least valuable. Pain (*n* = 9, 25%) appeared most frequently as one of the top three valuable attributes. Falls was second (*n* = 8, 22%) and mobility (*n* = 6, 17%) and physical activity (*n* = 6, 17%) were tied for third. However, providers considered mobility and physical activity (exercise) to be similar, and if combined, it would become the attribute most frequently appearing in the top three (*n* = 12, 33%). When asked to select the overall most important attribute, falls (*n* = 3; 25%), pain (*n* = 3; 25%), and mobility (*n* = 3; 25%) were tied for first.

Providers had difficulty choosing the least valuable data points, with some choosing to not select any as least valuable. Among those that did, driving appeared as a bottom three selection (*n* = 5; 23%) most frequently, followed by fatigue (*n* = 4, 18%). Both attributes, along with mood, were also ranked as the overall least important attributes, each selected twice (17%). Several providers were intrigued by a cognitive test, but did not consider a smartwatch an ideal device to administer such a test and therefore, would not trust the results. A summary of the rankings is in Table 2.

**Table 2:** Data rankings^a^.

Data point	Geriatricians (freq)	Surgeons (freq)	Anesthesiologists (freq)	Phys. Med and rehab (freq)	Nurses (freq)	Total freq (%)
Top 3 valuable ranking						
Pain	2	4	2	–	1	9 (25%)
Falls	4	2	1	1	–	8 (22%)
Mobility	1	3	1	1	–	6 (17%)
Physical activity (exercise)	1	2	2	1	–	6 (17%)
Hydration	2	1	–	–	1	4 (11%)
Medication adherence	1	–	–	–	1	2 (6%)
Fatigue	1	–	–	–	–	1 (3%)
Bottom 3 least valuable ranking						
Driving	–	3	1	1	–	5 (23%)
Fatigue	1	1	1	1	–	4 (18%)
Cognition testing	1	1	1	–	–	3 (14%)
Medication adherence	1	2	–	–	–	3 (14%)
Hydration	–	2	–	1	–	3 (14%)
Mood	1	1	–	–	–	2 (9%)
Mobility	–	–	–	–	1	1 (5%)
Physical Activity (exercise)	–	–	–	–	1	1 (5%)

^a^Not all providers selected three attributes as most/least valuable.

Providers were also shown data visualization samples, including line charts, pie charts, bar graphs, and radial gauges. Line graphs were the most popular (*n* = 6, 50%) because they easily conveyed longitudinal data that could be tracked over time. Bright color schemes were also favored, because it was important that data could be quickly interpreted. After viewing red, yellow, and green line charts, a geriatrician said the color-coding “helps to get an instant idea of how things have changed”.

##### Theme 4a: Baseline data needed

Providers expressed a need for baseline data to serve as a comparison. A surgeon commented, “We try to get [data] before and after, baseline and follow up. It’s really for us to show we’re doing a good job and to ensure that we’re hitting a target”. Currently, a surgeon collects data using paper and pencil, and then it is entered into a computer. Baseline data were particularly important for surgeons because it was the only way of knowing whether patients were closer to reaching the level of function previous to the procedure. A nurse also saw the need for baseline data because, “If we don’t have data before, we don’t know the ideal goal or range for that patient to be in. It becomes a guess”.

## Discussion

Although the benefits of patient-generated health data have been recognized at the policy level^9^, and health system leaders believe such data are important for collecting biometric (weight, blood pressure, and blood glucose) and patient activity data (exercise and nutrition)^28^, successful adoption of PGHD_SW ultimately depends on the actual users of the data. Our in-depth qualitative interviews with providers from diverse specialties found that PGHD_SW was considered to have the potential to be highly valuable because it enabled access to information about patients that was traditionally unattainable. In addition, PGHD_SW can contribute to more productive discussions between patients and providers, in both the in-patient and out-patient setting.

Our findings indicate that PGHD_SW may be unique compared to other types of patient-generated health data. Other methods of capturing patient data, like PROMIS measures, typically excludes patients with low literacy^29^, while PRO data often requires significant administrative burdens to both patients and providers^30^. Data from smartwatches allow the patient, instead of the provider, to take ownership of generating and capturing data^31^. We found that few providers were concerned about PGHD_SW adding to workloads. Rather, providers in our study viewed PGHD_SW as a means of reducing workload due to the ability of monitoring patient’s behaviors, which inform decision-making and can lead to reductions in patient and healthcare burden. Administrative burden can potentially be reduced since PGHD_SW can integrate with electronic records, and due to PGHD_SW’s continuous monitoring, it can be more accurate and portable than collecting sensor data using other devices, like smartphones.

Similar to previous studies which determined the importance of developing practice workflows^32^, our findings indicate that PGHD_SW should be tailored by medical specialization based on factors like patient population, medical setting, and procedure. Moreover, we discovered that providers view PGHD_SW as an opportunity to collaborate with patients. Patient engagement, and attention to maintaining healthy behaviors, could be enhanced if providers partner with patients about the meaning and context of their data^33^. Furthermore, it may be possible to improve the patient-provider relationship if PGHD_SW is integrated into interactions^34^. However, patients and providers might have different perceptions about the value of PGHD_SW, therefore, it is important to align patient and provider perceptions^35^.

Another patient-provider interaction issue that arose from our study were reservations related to trusting the data generated from smartwatches. Previous studies have shown that providers sometimes have difficulty believing patients with surprising or unusual symptoms^36^ and assign a label of “unreliable” to their patient^37^. However, PGHD_SW has the opportunity to quell suspicions since most data are passively collected^22^ and therefore, may be more resistant to patient modification. Similarly, some providers expressed the need for baseline data. Such data are attainable since collection of PGHD_SW begins as soon as a device is used.

Pain, falls, mobility, and physical activity were the most preferred and relevant data points, but providers saw value in all of the other data points presented. However, cognitive assessment triggered concerns about how the test could be administered using a smartwatch. Currently, technology to measure cognitive function has been developed and tested using smartphones^38–40^, but such tests are still in the developmental phases for wearable devices^41,42^.

Diffusion of Innovations’ framework proved helpful in contextualizing findings from the study, discovering that PGHD_SW has advantages over other types of patient-generated data and can be compatible with current workflows for a variety of medical specialties. For instance, upon viewing samples of output data, providers suggested that PGHD_SW be integrated with electronic medical records. Experts agree that the meaningful use of patient-generated health measures in clinical practice should begin with willing providers, rather than a large-scale implementation^43^. The trialability attribute from DoI would allow for a phased implementation using EHRs, in a tested environment. Thus far, PRO data have been successfully implemented into the EHR to provide quantitative, objective data regarding patients’ health status^44^. Additionally, DoI also promotes the use of opinion leaders, or champions, to advocate for the innovation^24^. Opinions leaders can be identified during the testing phase can accelerate the use of PGHD_SW and can lead to more providers embracing use of the data. Other studies have demonstrated that physicians under the influence of an opinion leader were more likely to adopt an innovation than physicians not under an opinion leader’s influence^45,46^. Other aspects of DoI will also be relevant to the adoption of PGHD_SW, such as the observability attribute once a prototype is developed, and the innovation-decision process^24^, during the implementation phase.

Limitations to our study include only focusing on perceptions of anticipated use, the small sample size and possible selection bias, in that providers volunteered for the study. Also, due to the small sample size, results cannot be generalized and reflect perceptions among other providers of the same specialty. Moreover, the feasibility of data-driven care largely inhabits the field of primary care. Our study focused on secondary care, but greater attention on this topic should be targeted towards primary care.

In addition to primary care, we plan to focus on other specialties to understand how providers manage the nuances involved with measurement specific to their specialty. We will also seek opinions and attitudes from patients about their perceptions of PGHD_SW collection. Ultimately, an application will be developed to enable seamless integration of PGHD_SW into electronic health records.

## Methods

### Sample and design

This study was conducted at UF Health, an 852-bed level I trauma center located in Gainesville, Florida. This study was approved by University of Florida Institutional Review Board by IRB 201801446, and all methods were performed in accordance with the relevant guidelines and regulations. Informed consent was obtained from subjects before enrollment in the study. A combination of purposive and convenience sampling^47^ was utilized to attain as many participants as possible from a wide variety of specialties. The goal was to obtain perspectives from providers in both the in-patient and out-patient setting. Providers were recruited from internal medicine, family medicine, geriatric medicine, nursing, surgery, rehabilitation, and anesthesiology.

An overview of the study was presented at periodic team meetings by members of the research team. Those interested in participating were sent an email describing the study. Additionally, individual providers who may not have been present at meetings were targeted through email because of their expertise and potential interest in participation. Providers interested in participating were scheduled for an interview in a private office.

A semi-structured interview guide was designed to gauge providers’ perceptions of PGHD_SW and the way data should optimally be presented. To construct the guide, four members of the research team (JA, PR, TM, MR) wrote an initial set of questions and then vetted them to align with the goals of the study. After modifications, the research team reached consensus on the structure and content of the questionnaire. The semi-structured interview guide consisted of 15 main questions, but was written with flexibility, to allow for follow-up questions based on individual responses^48^. Selected questions from the interview guide are in Box 1. In addition, sample data visualization graphs and possible data points were presented to the interviewees to collect preferences. All interviews were conducted by the first (JA) and third author (MR) and were audio recorded and subsequently transcribed. Informed consent was reviewed and written consent was received before interviews were conducted.

Box 1 Sample interview guide questions • What are your views about viewing data generated by your patients via smartwatches?

• How often would you like to receive summaries of patient data?

• How do you think PGHD from smartwatches will contribute to patient care?

• How important is PGHD to you, compared with other medical information you interpret, such as blood tests, scans, X-rays…etc?

• How will you modify your communication with patients/caregivers now that they can enter and view PGHD data?

### Analytical process

Transcripts of interviews underwent independent, open coding by the first (JA) and third authors (MR) using the constant comparative method^49^. This method helps to reduce the data to concepts^50^ through an iterative and inductive process of reducing the data through constant recoding^51^. Using the open codes, we created a comprehensive codebook. Interviews continued during data analysis until no new themes emerged and saturation was achieved^52^ through recurrence, repetition, and forcefulness of the data^53^. Transcripts were then re-read using the codebook and reviewed to identify emerging insights^54^. Codes were condensed and developed into preliminary themes, guided by DoI’s attributes of an innovation. However, one of the attributes, observability, was not included since fully functioning PGHD_SW was not yet applicable at the time of the study. Discrepancies among the research team were resolved by revisiting the codebook and through discussions by the entire research team until consensus was reached^55^. To validate our conclusions, numbers were used to provide quantitative confirmation to make claims more precise^56^. Quantitative counts have several advantages in qualitative research, such as enabling the identification and characterization of diverse perceptions, and providing evidence for interpretations^57^.

### Reporting summary

Further information on research design is available in the Nature Research Reporting Summary linked to this article.

## Supplementary information

Reporting Summary

## Data Availability

Selected quotes from participants are included in the article; supplementary tables include data about providers’ preferences. The full dataset is available from the corresponding author upon reasonable request.
